# Motivation by, Perceived Quality of and Satisfaction with Sports Services among Young Athletes: A Psychological Approach

**DOI:** 10.3390/children9101476

**Published:** 2022-09-27

**Authors:** Antonio Aznar-Ballesta, Eva María Peláez-Barrios, Alicia Salas-Morillas, Mercedes Vernetta

**Affiliations:** 1 Research Group “Science and Sport” SEJ 470, Faculty of Sports Sciences, Department of Physical and Sports Education, University of Granada, 18071 Granada, Spain; 2 Research Group “Analysis and Evaluation of Physical-Sports Activity” CTS 171, Ministry of Education and Sports, Junta de Andalucía, 41092 Sevilla, Spain; 3 Research Group “Analysis and Evaluation of Physical-Sports Activity” CTS 171, Department of Dance, Acrobatics and Circus, Alicia Alonso University Institute, 28942 Madrid, Spain; 4 Research Group “Analysis and Evaluation of Physical-Sports Activity” CTS 171, Faculty of Sports Sciences, Department of Physical and Sports Education, University of Granada, 18071 Granada, Spain

**Keywords:** young athletes, team sports, sports service, extracurricular physical activity, motivation, quality, satisfaction

## Abstract

The aim was to assess the psychological approach of young athletes to sports services in terms of motivation, perceived quality and satisfaction. A total of 307 adolescents (55.7% male and 44.3% female) between 12 and 18 years old participated. Three questionnaires were applied: an ad hoc questionnaire, the Self-Report of Motivation to Practice Physical Exercise (AMPEF) and the Questionnaire for the Evaluation of Sports Services (EPOD2). A descriptive, cross-sectional study was conducted, applying regression analysis to determine the predictive nature of the factors in the questionnaires. Individual sports players and non-competitors rated the motivational factors lower than those who practised collective sports and competed, and the results were reversed with regard to the perceived quality of, satisfaction with and value of the service. They valued human and personal treatment more highly, correlating it moderately and positively with service value (r = 0.422 for the monitor and r = 0.442 for the organisation’s staff) and satisfaction (r = 0.43 for the monitor and r = 0.552 for the organisation’s staff). Satisfaction was a negative predictor of extrinsic motivation, and vice versa (β = −0.207 and β = −0.143). Young athletes, in general, have an orientation towards intrinsic motivation and therefore a low tendency towards sports dropout.

## 1. Introduction

Different studies indicate that physical-sporting activity (PSA) improves not only the physical state of the athlete but also the mental (psychological) state, combating stressful situations and improving self-esteem and socialisation [1,2,3,4,5,6,7]. Moreover, in socio-economic situations such as the current one, in which the moment one economic crisis ends, another starts, with consequent social tensions, sports can act as a palliative with respect to economic savings in public health and reduce the personal tensions resulting from the economic crises [8,9]. In spite of this, in its 2020 study, the World Health Organization (WHO) indicated that the rate of sports practice worldwide was still insufficient to improve the quality of life [10].

In relation to the above, because of the importance of PSA during adolescence, when healthy habits that last a lifetime can be created, there has been extensive study on what motivates people to take up PSA [11]. In this sense, different researchers have created and validated questionnaires capable of measuring the different reasons for practising sports [12,13,14,15]. Most of the questionnaires are based on Deci and Ryan’s [16] self-determination theory, which states that people feel motivated when they satisfy or want to develop their innate need for autonomy, competence and socialisation. The questionnaires also divide the types of motivation into intrinsic motivation and extrinsic motivation. in addition to segmenting the types of extrinsic motivation is further divided into external regulation, introjected, identified and integrated, and although introjected, identified and integrated are self-regulated by the athlete, there is an external influence that conditions them [17]. Even though there are a greater number of extrinsic regulations, people who have higher rates of intrinsic motivation will tend to have a more active and, therefore, healthier sporting life, favouring the intrinsic motivational state [18,19]. In this sense, encouraging autonomous motivation in the athlete by orienting motivation towards activities that promote self-efficacy will help the athlete continue PSA [20,21,22,23,24,25]. Therefore, it should be the coaches or monitors who first learn about the predominant types of motivation in their athletes and acquire specific training that includes the promotion of autonomy, which is a requirement for athletes and employers in the sports sector [26,27,28,29]. As a result of the industrialisation of the sector, there are already faculties of sports science in the United States that offer subjects, within their degrees, specialising in sales, for which it is necessary to know the reasons people approach PSA in order to offer the product that best suits the athletes [30].

Satisfaction with and the perception of quality in sports services will improve loyalty and encourage healthy habits [31]. The indices can be assessed through questionnaires [32,33,34] that can help to measure loyalty or future intentions [35]. Moreover, in view of the incipient ageing of the population, research is already being carried out on segmentation by age when it comes to managing the quality of services, taking into account that needs change depending on the age of the athlete: childhood, adolescence, adulthood or the elderly [36]. In this sense, the perception of quality can result in satisfaction and, together, both can encourage loyalty in sportspeople towards PSA [37,38]. Although users of sporting activities are satisfied with the services offered, they continue to demand quality, which is a determining factor in their satisfaction [39]. The measuring athlete satisfaction and providing sufficient information about the activity to be performed, among others [40]. In this sense, equipment and the quality of the environment (maintenance, noise, lighting, air quality, temperature) were also found to determine the general assessment of sports services [41]. Due to the high level of competition and the requirements of sportspeople attending the centres, it is advisable to obtain opinions through surveys in order to help managers to take measures based on the data obtained [42].

Therefore, taking into account the importance of continuity in the practice of healthy habits, such as PSA, it is necessary to establish extracurricular sports as a field of study, taking into account both the psychological factors that lead people to practice and the aspects to be assessed that form part of a sports service or the entity as a whole.

In relation to the above, the aim of the study was to evaluate what motivates young people, between 12 and 18 years of age, to take up sports, taking into account their manifestations and relating them to the perceived quality of, satisfaction with and general assessment of public, associative and private after-school sports services.

## 2. Material and Methods

### 2.1. Design and Participants

This non-experimental, descriptive, cross-sectional study involved 307 young athletes, between 12 and 18 years old (M = 14.78, SD = 1.72), in which 55.7% were male (n = 171) and 44.3% were female (n = 136). The participants were selected through non-probabilistic, purposive sampling. All participants were involved in some form of PSA, mainly at public centres, clubs, associations and/or (to a lesser extent) private centres. In the selection of the entities we have taken into account all those existing in the district of Maracena: volleyball, athletics, dance, basketball, handball, billiards, mountain biking, caving, American football, soccer, rhythmic gymnastics, karate, wrestling, taekwondo, volleyball, urban sports and private gyms dedicated to CrossFit in the district of Maracena in the province of Granada (Spain). Participation was voluntary, and anonymity in participation was facilitated. The study complied with the ethical principles for research involving human subjects set out in the Helsinki Declaration of 1975 and was approved by the Research Ethics Committee of the University of Granada (no: 2286/CEIH/2021).

### 2.2. Instruments

To analyse the profile of the young people, an ad hoc questionnaire was drawn up with socio-demographic questions on gender, year of study (age) and the type of sport (whether the sport they played was an individual or a team sport and whether it was competitive or not). The values of the questionnaire were interpreted using the mean and standard deviation values.

*Self-Report of Motivation to Practice Physical Exercise* (AMPEF), using the Spanish adaptation created by Capdevila et al. [43], has been considered valid and reliable for measurement [43,44]. It consists of 48 practice motives (items) grouped into 11 factors: (1) weight and body image, (2) fun and well-being, (3) prevention and positive health, (4) competition, (5) affiliation, (6) muscular strength and endurance, (7) social recognition, (8) stress management, (9) agility and flexibility, (10) challenge and (11) health emergencies. Cronbach’s alpha values obtained by Capdevila et al. [43] were, respectively, 0.92, 0.71, 0.84, 0.90, 0.87, 0.83, 0.87, 0.85, 0.84, 0.85, 0.85 and 0.54. However, in this study, the values were respectively, 0.88, 0.77, 0.83, 0.81, 0.73, 0.82, 0.75, 0.77, 0.77, 0.77, 0.74 and 0.68. The 11 factors could also be separated into two factors: intrinsic motivation and extrinsic motivation. The factors fun and well-being, muscular strength and endurance, challenge, competition, agility and flexibility, affiliation and prevention and positive health belonged to the first block and health emergencies, weight and body image, social recognition and stress management belonged to the second [19]. The responses to the questionnaire were measured on a 10-point Likert-type scale, ranging from 0 = strongly disagree with the statement to 10 = strongly agree with the statement. The values of the questionnaire were interpreted by the mean and standard deviation values of each factor, which consisted of several items. The closer the factor means were to a score of 10, the higher the motivation in the particular motivational aspect.

Finally, in order to measure the perceived quality of, satisfaction with and value of the service in sports entities for PSA, the *Questionnaire for the evaluation of sport services* (EPOD2) was used [45]. It consists of 25 items and 8 factors: (1) monitor/coach, (2) facilities, (3) sports material, (4) activities, (5) communication, (6) organisation staff, (7) satisfaction in relation to the organisation and activity and (8) quality/price ratio of the activity or value of the service. The first 6 factors belong to the area of perceived quality evaluation with Cronbach’s alpha of. 89, factor 7 corresponds to service satisfaction with a Cronbach’s alpha of 0.86 and factor 8 corresponds to a service value (only 1 item) [45]. For the present study, Cronbach’s alpha values were 0.89 and 0.88 for the same factors. It was measured on a Likert scale from 1 to 5 (1 = strongly disagree, 2 = disagree, 3 = agree, and 4 and 5 = strongly agree). The questionnaire values were interpreted using the mean and standard deviation values of each factor, which consisted of several sub-factors in the case of perceived quality and, in turn, of several items, except in the case of service value. The closer the factor means were to a score of 5, the higher the perceived quality, satisfaction or service value, as the case may be.

### 2.3. Procedure

We contacted coaches and athletes aged between 12 and 18 years old from different sports entities (public, associative and private) who were in some year of compulsory secondary education (ESO) or baccalaureate. The questionnaire was completed in paper format and was provided to the respondents in person before or after they had started their activities or training during the months of November and December of 2021, having been previously informed of the objectives of the study and with the consent of the families and management teams of the different clubs and centres.

### 2.4. Statistical Analysis

Data were analysed using IBM SPSS Statistic 26 (Chicago, IL, USA). Continuous variables were presented as the mean, the standard deviation and 95.0% confidence interval and categorical variables as frequencies. The normality of the variables was analysed using the Kolmogorov–Smirnov (K–S) test. A non-parametric analysis was chosen when a non-normal distribution was observed. For the main analysis with quantitative variables, the means were compared using the Mann–Whitney U test and if the independent variable had more than two categories, the Kruskal–Wallis H test. Spearman’s correlation was used to test the relationship between the satisfaction and motivation dimensions of the PSA questionnaire. In addition, the Chi2 test or Fisher’s test was used for qualitative variables. Stepwise multiple regression analysis was used to explore which variables could explain the variation in the dependent variable. The requirements for including an independent variable in the multiple regression analysis were as follows: (1) the correlation coefficients between the dependent and independent variable were significant (to see if an independent variable partially explains a dependent variable, both variables must be significant to each other) and (2) the correlation coefficients between the independent variables were equal to or less than 0.70 (the association between two independent variables should be less than this value to avoid collinearity and therefore avoid introducing variables that explain the same thing). Statistical significance was set at *p* < 0.05.

## 3. Results

The sample of the present study consisted of 307 young athletes aged 12-18 years (M = 14.78, SD = 1.72), where 55.7% were male (n = 171) and 44.3% female (n = 136). All of them participated in individual and collective extracurricular DFA in public or private entities or in sports clubs for training and competitive purposes.

Table 1 shows the mean scores and the standard deviation of the factors of the AMPEF questionnaire in relation to social variables and extracurricular sports practice.

Statistically significant gender differences were found in the following factors: weight and body image, prevention and positive health, competition, affiliation, strength and endurance, social recognition and health emergencies. In this sense, the biggest differences between boys and girls were in terms of competition and social recognition. There were no significant differences in the remaining factors, as in this group of factors, the highest (fun and well-being) and the lowest (health emergencies) scores were found for both sexes, where boys’ scores were always higher than girls’ in all factors.

When factors were related to the type of sport performed (individual or collective), significance was found in weight and body image, prevention and positive health, competition, strength and endurance, social recognition and health emergencies. Scores were always higher in all factors for athletes playing team sports. The biggest difference was found in the competition factor, with team sports had higher scores.

In terms of the academic year attended (age), significant differences were found in the factor agility and flexibility when students in the first cycle of ESO were compared with those in the baccalaureate (*p* = 0.038). Students in the first cycle of ESO scored higher on all factors except stress control and health emergencies, which were rated higher by those in the baccalaureate but there are no significant differences.

In relation to whether it was a sport or competitive PSA or not, significant differences were found in the following: weight and body image, prevention and positive health, competition, affiliation, strength and endurance, social recognition, stress management, agility and flexibility, challenge and health emergencies. Related to the above, adolescents who competed were more motivated and the largest differences in scores with non-competitors were observed in the factors competition and social recognition.

Table 2 shows the mean scores of and standard deviations in the factors of the EPOD2 questionnaire in relation to social variables and extracurricular sports practice.

Statistically significant differences were established with respect to sex for the following factors: perceived quality of sports facilities; perceived quality of facility staff; and satisfaction with and value of the service, both with the same significance (*p =* 0.019). Boys generally rated lower scores than girls. Within factors referring to service quality, facilities scored the lowest among both sexes, while monitor/coach scored the highest among boys and organisation staff scored the highest among girls.

Regarding whether the sport practised was an individual or a team sport, there were significant differences in all factors, except for perceived quality in communication, in which team sports players showed lower ratings than individual sports players in all factors.

The perceived quality of the monitor/coach was found to be significant between the first and second cycles of ESO (*p* = 0.006), and the perceived quality of the activities was found to be significant between the first cycle of ESO and baccalaureate (*p* = 0.014).

In relation to whether the practice of PSA was competitive or not, all factors were found to be significant except the perceived quality of communication.

**Figure 1:**
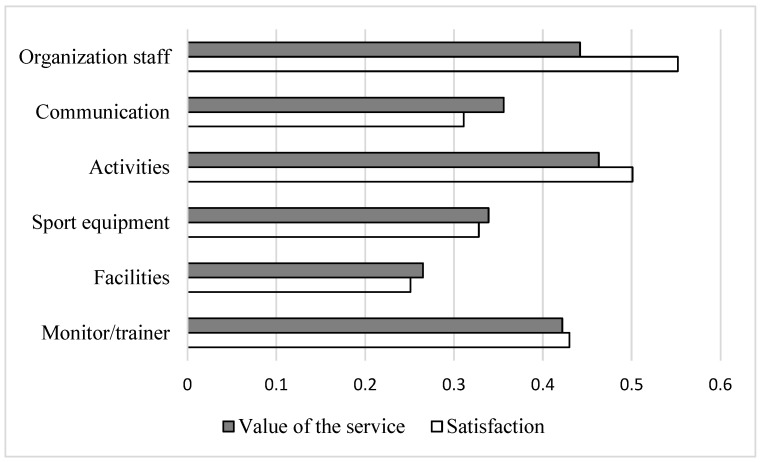
Bivariate correlations (Spearman) between the sub-factors of perceived quality and overall service value and satisfaction.

It was also possible to detect the degree of correlation between the components that intervene in any sports entity and the satisfaction with and general assessment of the services used by the athletes, being statistically significant in all cases, in order to determine which were the most decisive when it came to evaluating the whole and part of their emotional states (Figure 1).

## 4. Regression Analysis

Table 3 shows the correlations between the AMPEF and EPOD2 factors.

All significant correlations were low or very low, except for the correlations of the factors of the questionnaires themselves (AMPEF and EPOD2), with ratings ranging from moderate to high.

Table 4 shows the results of the test conducted to determine the degree or weight of the independent variables in the different models.

Bivariate correlations were recorded for five dependent variables (Table 3), through which five different regression models were obtained between them, of which only the models in which the predictor variables did not belong to the same questionnaire (extrinsic motivation and satisfaction. Table 4) were analysed, explaining the models below.Analysis of variance (ANOVA) revealed that the explained variance was higher than the unexplained variance for extrinsic motivation (AMPEF) (F = 113.190; *p* < 0.001). There was a positive effect of the variable “intrinsic motivation” (AMPEF) and a negative effect of the variable “satisfaction” (EPOD2), evaluated on the basis of the main variable. The model explained 42.7% of the overall unadjusted variance and 42.3% of the overall adjusted variance in extrinsic motivation. Thus, regression analyses revealed that intrinsic motivation and satisfaction were significant predictors and when combined, explained 42.3% of the variance in the extrinsic motivation scores (adjusted R^2^ = 42.3%; F = 22.498; *p* < 0.001), with the “intrinsic motivation factor being the maximum predictor (Table 4).

Regarding satisfaction (EPOD2) (ANOVA, F = 96.473; *p* < 0.001), the model explained 48.9% of the variance, resulting in 48.3% after correction combining the factors “service value” and “perceived quality” with a positive relationship and “extrinsic motivation” with a negative relationship (adjusted R^2^ = 48.3%; F = 12.098; *p* < 0.001), with the factor “perceived value” being the maximum predictor (Table 4).

## 5. Discussion

The purpose of the study was to evaluate the ways in which the motivation of people between 12 and 18 years of age manifests itself, as well as the perceived quality, satisfaction and general assessment, establishing possible relationships between them, in public, associative and private after-school sports services.

Ranking the mean ratings of the 11 factors of the AMPEF questionnaire, the highest rated factor was fun and well-being, followed by challenge, muscular strength and endurance, competition, affiliation, positive health prevention, agility and flexibility, stress management, weight and body image, social recognition and health emergencies. Therefore, taking into account that the first seven factors coincide with assessments of intrinsic motivation, they corroborate the results of the studies by Galan-Lopez et al. [19] and Feliz de Vargas Viñado and Herrera Mor [21], being able to determine that extrinsic motivation has less influence extracurricular PSA by adolescents, although motivational aspects associated with social recognition are increasingly being detected [46]. In this sense, it is considered that the greater influence of intrinsic motivation was due to the fact that they are sports modalities to which adolescents enroll voluntarily and, with respect to the search for social recognition may be due to the very important weight of the sports industry in the media [47].

Significant differences were found in 7 of the 11 factors of the AMPEF questionnaire with respect to gender, unlike the study by Fradejas-Medrano and Espada-Mateos [20], in which no significant differences were found. However, six factors were found with differences in the study by Galan-Lopez et al. [19], in which the factors weight and body image, competition, strength and muscular endurance and social recognition coincided with those in the present study. Similarly, the factors weight and body image and strength and muscular endurance in the research by Colunga-Rodríguez et al. [48] coincided with those in the present study. However, the study by Domínguez Alonso et al. [44] obtained significant differences in seven factors, of which competition, affiliation, strength and endurance, social recognition and health emergencies coincided. Boys always indicated higher ratings, as in other research [19,44,46], indicating that the concern for body image, historically assigned to girls, is being increasingly valued by boys [49]. However, in a recent study in the Netherlands, it was girls who rated body image the highest, with boys giving the highest score to competition [15], or in Norway, where the highest scores were assigned by girls to factors related to intrinsic motivation [50], as policies, neighborhoods and culture differ from place to place. The above data show that sport motivations differ according to the environment in which they are developed [51]. That said, what does seem to be common in different studies is that the factor “fun and well-being” tends to be the most highly rated by both sexes [52].

Team sports players showed higher motivation indices in all factors compared to individual sports players, coinciding with the study by Fradejas-Medrano and Espada-Mateos [18]. In another context, van Lankveld et al. [15] scored higher on mastery, enjoyment, affiliation, competition and expectation of others, whereas in individual sports, it was the motives of seeking better physical condition, psychological condition and appearance. However, individual sports are often associated with intrinsic motivation [22,23], although in our case, no such influence was detected over group sports, which could be due to the fact that most of the sample played football, the favourite sport of Spaniards. This explains why, depending on the motives and personality of PSA practitioners, different types of sports are chosen [53].

Regarding the training cycle (age), Fradejas-Medrano and Espada-Mateos [18] found no differences in motivation between students in the first cycle and those in baccalaureate, as in our study, with the exception of the factor “agility and flexibility”. Domínguez Alonso et al. [44] found differences in stress control between students in baccalaureate and those in first cycle, as well as between the students in the two courses that make up the first cycle of ESO, where the highest scores were in the second cycle, differing from the present study, where all scores were higher in the first cycle and in baccalaureate. In health emergencies, there was agreement in significance with respect to our study. However, the study by Colunga-Rodríguez et al. [48] found a significant association between age and all the factors of the AMPEF questionnaire, with the factors with the highest scores, i.e., competition and social recognition, coinciding with those of the study by Portela-Pino et al. [54], where, with the passage of time, not only greater motivation was observed, but also a greater number of barriers to practice. In this sense, it seems that the generalisation of the results has no place in this aspect, as competition and appearance in relation to increasing age may also be weakly associated [15].

Athletes in competitive sports showed higher rates of motivation in all factors compared to non-competitors, with competition and social recognition being the main motives, being in line with the work by van Lankveld et al. [15]. However, although there is research indicating the existence of an extrinsic motivational preference in competitive sports, the higher scores of the non-competitors in the “competition” and “social recognition” factors were more consistent with the work of Escamilla-Fajardo et al. [55]. However, although there is research indicating the existence of an extrinsic motivational preference in competitive sports, the highest scores the competitors in the study assigned were to intrinsic motivational factors. In relation to the above, it seems that competition in the athletes who are the subject of this study is one of the reasons for their personal enjoyment without being more closely related to motivational aspects external to the athlete and, among other reasons, to the great television repercussions that exist around the broadcasting of competitions on television.

In general, the highest scores in our study related to perceived quality were assigned in order to the following sub-factors: monitor, facility staff, activities, material, communication and facilities. In this sense, it should be borne in mind that it is people who have the highest ratings, so it seems to be that a pattern has been found in which when there are a majority of athletes under the direction of municipal sports, the results are associated with social learning and emotional well-being, i.e., that which is provided by the sportspersons themselves [38], or the human factor, as is the case in the studies by Nicolás-López and Escaravajal-Rodríguez [56], Robles et al. [57] and Castillo-Rodriguez et al. [37]. However, when the athletes were users of private centres, the scores were positively reversed towards the equipment and the conditions in which the sports space is located. [41,58]. The fact that workers in public sports associations or services show higher satisfaction rates than those in the private sector may have to do with the satisfaction ratings of those in the private sector [59]. This may be related to the above-mentioned evaluations, in addition to the fact that private entities are committed to a continuous renovation of materials, while public services, due to the bureaucratic complexity, tend to have greater difficulties.

With respect to gender, there were significant differences in perceived quality in two of the five sub-factors (facilities and organisational staff), with girls generally scoring higher. In this sense, the study by Nuviala Nuviala et al. [60], with similar results, rated the quality and value of the service positively, thus predicting satisfaction. However, in different settings, higher ratings were also found for boys [57]. Even so, it seems that the scores assigned by the girls may be due to the fact that the activities they practice the most are in closed facilities that require special comfort and, in addition, to access them, they always have to enter through areas where the organisation’s staff must be present to control them.

Taking into account that boys prefer collective sports and girls prefer individual/choreographic sports [61,62], athletes in individual sports or activities show higher levels of satisfaction or enjoyment due to the greater assignment of responsibility for their results, with intrinsic objectives, thus generating greater resistance to adversity [63]. In line with individual or group sports, competitive or non-competitive activities are found, given that non-competitive activities are usually more associated with individual sports, thus obtaining the highest scores.

Relating the training cycle (age) to satisfaction, service value and perceived quality, several studies indicate that the higher the age, the higher and better the perception of quality [37,57,60], probably due to the maturity of sportsmen and women and the importance given to value for money over the years, as teenagers tend to pay for their leisure and free time activities as they get older. The data from the current study coincided with these results in terms of all factors except sports equipment, satisfaction and value of service, all without significant differences, coinciding with the study by Romanova and Sollar [64], in which the highest ratings were given at the youngest ages.

Regarding the predictor character obtained from the regression analyses of the factors of the two validated questionnaires, coincidences were found around the three constructs of the EPOD 2 [37,57]. Furthermore, the fact that satisfaction is a negative predictor of extrinsic motivation and vice versa makes us reflect on the suitability of linking the psychological and cognitive processes of sports learning with intrinsic motivation in order to prevent athletes from abandoning sports [18,19,63], as it is understood that a person with high rates of external influences when practising PSA will develop or look for easy excuses not to practise more, thus self-promoting dissatisfaction during service consumption.

Among the limitations of the study is the use of an indirect method using questionnaires, with the complexity involved in the process of administering them, as some heads of organisations or DFAs expressed their disagreement with the length of the questionnaires, as they were completed before the sessions.

The study has several practical implications since, by knowing the direction of athletes’ motives regarding sports, coaches can predict and treat demotivation caused by high levels of extrinsic motivation.

The significance of this research is associated with the fact that the managers of the different sports entities can combat abandonment of sports and promote customer loyalty by boosting intrinsic motivation and improving the quality of their services depending on the type of management (public/private).

In future research, it would be useful to know what quality related to sports services each athlete prefers and how each athlete perceives a quality, creating a ranking of primary needs to be satisfied when accessing a sports service.

## 6. Conclusions

In the light of the results, it can be concluded that the adolescents in this study were motivated by and satisfied with the services offered at the sports facilities. Boys showed higher rates of motivation, and girls had a better perception of the services in terms of quality, satisfaction and value.Those who played individual sports, as well as those who did not compete, rated the motivational factors lower than those who played group sports and competed, and the results were reversed for the perceived quality of, satisfaction with and value of the service.In addition, adolescents valued the human and personal treatment more, correlating moderately and positively with the value of the service and satisfaction, the latter being a negative predictor of extrinsic motivation and vice versa.

## Figures and Tables

**Table 1:** Relationship between the factors of the AMPEF questionnaire and socio-sporting variables.

Variables		Man (n = 171)		Woman (n = 136)		Individual (n = 127)		Collective (n = 180)		^1^ First Cycle ESO (n = 114)			^2^ Second Cycle ESO (n = 121)		^3^ Bach. (n = 72)			Comp. (n = 252)		No Comp. (n = 55)	
		M	DT	M	DT	M	DT	M	DT	M	DT		M	DT	M		DT	M	DT	M	DT
Weight/image corp.		5.80	2.45	4.80	2.86	4.70	2.95	5.82	2.37	5.56	2.88		5.20	2.58	5.28		2.53	5.63	2.53	4.09	2.98
*p*	U or H	0.004		9387.50		0.002		13,827		>0.05		^1^-^2^ 1.748		^1^-^3^ 2.036		^2^-^3^ 0.080		<0.001		4893.50	
Fun and well-being		8.54	1.44	8.37	1.60	8.43	1.57	8.49	1.47	8.64	1.39		8.36	1.59	8.37		1.55	8.55	1.41	8.08	1.87
*p*	U or H	>0.05		10,986		>0.05		11,595.50		>0.05		^1^-^2^ 1.883		^1^-^3^ 2.132		^2^-^3^ 0.005		>0.05		6101.50	
Prevention and health +		7.28	2.12	6.49	2.29	6.49	2.54	7.24	1.93	7.09	2.25		6.83	2.26	6.83		2.16	7.14	2.11	5.95	2.49
*p*	U or H	0.001		9143		0.030		13,092.50		>0.05		^1^-^2^ 0.945		^1^-^3^ 1.350		^2^-^3^ 0.020		0.001		4978.50	
Competition		8.18	1.90	5.93	2.88	5.90	3.04	8.08	1.84	7.33	2.52		7.23	2.71	6.86		2.68	7.94	1.86	3.68	2.83
*p*	U or H	<0.001		5975.50		<0.001		16,398.50		>0.05		^1^-^2^ 0.012		^1^-^3^ 1.809		^2^-^3^ 1.354		<0.001		1624	
Affiliation		7.33	2.15	6.75	2.22	6.89	2.36	7.20	2.07	7.27	2.15		6.95	2.17	6.97		2.32	7.33	1.95	5.89	2.81
*p*	U or H	0.013		9709		>0.05		12,104.50		>0.05		^1^-^2^ 1.460		^1^-^3^ 1.513		^2^-^3^ 0.035		<0.001		4927	
Strength and endurance		7.92	2.07	6.50	2.55	6.62	2.83	7.77	1.91	7.42	2.58		7.27	2.81	6.59		2.30	7.64	2.13	5.71	2.88
*p*	U or H	<0.001		7615.50		0.002		13,829.50		>0.05		^1^-^2^ 1.724		^1^-^3^ 2.537		^2^-^3^ 0.212		<0.001		4220	
Social recognition		5.80	2.45	3.86	2.44	4.10	2.66	5.53	2.44	5.22	2.86		4.93	2.50	4.49		2.41	5.49	2.42	2.39	1.94
*p*	U or H	<0.001		6680.50		<0.001		15,054.50		>0.05		^1^-^2^ 0.692		^1^-^3^ 3.143		^2^-^3^ 1.287		<0.001		2282.50	
Stress management		5.93	2.89	5.33	3.17	5.38	3.21	5.87	2.88	5.53	3.09		5.46	2.99	6.22		2.95	5.95	2.89	4.38	3.34
*p*	U or H	>0.05		10,320		>0.05		12,335.50		>0.05		^1^-^2^ 0.030		^1^-^3^ 3.371		^2^-^3^ 3.017		0.001		5015.50	
Agility/flexibility		6.41	2.61	6.22	2.85	6.24	3.04	6.39	2.47	6.68	2.79		6.25	2.64	5.90		2.68	6.57	2.57	5.20	3.09
*p*	U or H	>0.05		11,311.50		>0.05		11,356		>0.05		^1^-^2^ 2.060		^1^-^3^ 4.547		^2^-^3^ 0.915		0.002		5101	
Challenge		7.66	2.00	7.33	2.21	7.20	2.39	7.74	1.85	7.60	2.30		7.45	2.03	7.50		1.91	7.85	1.81	6.00	2.61
*p*	U or H	>0.05		10,714.50		>0.05		12,597		>0.05		^1^-^2^ 1.125		^1^-^3^ 1.580		^2^-^3^ 0.002		<0.001		4021	
Health emergencies		2.43	2.67	1.76	2.35	1.45	2.36	2.61	2.58	2.17	2.55		1.90	2.38	2.45		2.83	2.33	2.56	1.22	2.30
*p*	U or H	0.015		9810.50		<0.001		14,795.50		>0.05		^1^-^2^ 0.567		^1^-^3^ 1.717		^2^-^3^ 1.698		<0.001		4870	

AMPEF: Self-report of motivation to practice physical exercise; ESO: compulsory secondary education; M: mean; SD: standard deviation; n: number of persons in the sample; corp.: corporal; +: positive; Bach: baccalaureate; Comp: competitive; *p*: Statistical significance; U: Mann-Whitney U; H: Kruskal Wallis H (year of study); ^1^-^2^: Kruskal Wallis H value between first cycle ESO and second cycle ESO; ^1^-^3^: Kruskal Wallis H value between first cycle ESO and baccalaureate; ^2^-^3^: Kruskal Wallis H value between second cycle ESO and baccalaureate.

**Table 2:** Relationship between the factors of the EPOD2 questionnaire and socio-sportive variables.

Variables		Man (n = 171)		Woman (n = 136)		Individual (n = 127)		Collective (n = 180)		^1^ First Cycle ESO (n = 114)			^2^ Second Cycle ESO (n = 121)		^3^ Bach. (n = 72)			Comp. (n = 252)		No Comp. (n = 55)	
		M	DT	M	DT	M	DT	M	DT	M	DT		M	DT	M		DT	M	DT	M	DT
Monitor/coach		4.60	0.65	4.64	0.57	4.85	0.29	4.45	0.73	4.50	0.68		4.69	0.56	4.68		0.60	4.57	0.66	4.82	0.31
*p*	U or H	>0.05		11,722		<0.001		8109		0.019		^1^-^2^ 7.150		^1^-^3^ 7.879		^2^-^3^ 0.378		0.05		7988.50	
Facilities		3.84	0.97	4.01	1.07	4.35	0.74	3.61	1.07	3.83	1.03		3.89	1.02	4.10		0.95	3.80	1.02	4.46	0.76
*p*	U or H	0.039		13,203.50		<0.001		6611		>0.05		^1^-^2^ 0.199		^1^-^3^ 3.315		^2^-^3^ 2.089		<0.001		9731.50	
Sports equipment		4.18	0.85	4.29	0.87	4.59	0.60	3.97	0.92	4.31	0.73		4.21	0.87	4.13		1.01	4.13	0.88	4.68	0.53
*p*	U or H	>0.05		12,648.50		<0.001		6746		>0.05		^1^-^2^ 0.093		^1^-^3^ 0.239		^2^-^3^ 0.069		<0.001		9574	
Activities		4.39	0.61	4.49	0.55	4.67	0.34	4.27	0.66	4.32	0.64		4.47	0.55	4.55		0.51	4.39	0.61	4.66	0.37
*p*	U or H	>0.05		12,447		<0.001		7420.50		0.037		^1^-^2^ 3.370		^1^-^3^ 6.596		^2^-^3^ 1.032		0.004		8621	
Communication		4.03	0.86	4.03	0.84	4.14	0.78	3.95	0.88	3.91	0.95		4.07	0.79	4.14		0.76	3.99	0.85	4.20	0.84
*p*	U or H	>0.05		11,626.50		0.087		10,136.50		>0.05		^1^-^2^ 1.304		^1^-^3^ 2.254		^2^-^3^ 0.213		>0.05		8015	
Staff org.		4.43	0.79	4.67	0.55	4.79	0.47	4.35	0.78	4.45	0.79		4.57	0.65	4.60		0.63	4.46	0.74	4.85	0.37
*p*	U or H	0.009		13,397		<0.001		7579		>0.05		^1^-^2^ 0.940		^1^-^3^ 1.798		^2^-^3^ 0.217		<0.001		8967	
Satisfaction		4.66	0.58	4.79	0.51	4.93	0.19	4.57	0.67	4.72	0.58		4.74	0.54	4.68		0.54	4.68	0.59	4.90	0.26
*p*	U or H	0.019		13,105.50		<0.001		8044		>0.05		^1^-^2^ 0.008		^1^-^3^ 0.433		^2^-^3^ 0.352		<0.001		8351.50	
Value of the service		4.50	0.86	4.70	0.70	4.86	0.43	4.40	0.93	4.63	0.75		4.56	0.79	4.57		0.87	4.53	0.84	4.87	0.43
*p*	U or H	0.019		13,027.50		<0.001		8348.50		>0.05		^1^-^2^ 0.373		^1^-^3^ 0.389		^2^-^3^ 0.143		0.001		8400.50	

EPOD2: questionnaire of evaluation of sports services; ESO: compulsory secondary education; M: mean; SD: standard deviation; n: number of people in the sample; org: organisation; Bach.: baccalaureate; Comp.: competitive; *p*: Statistical significance; U: Mann-Whitney U; H: Kruskal Wallis H (year of study); ^1^-^2^: Kruskal Wallis H value between first cycle ESO and second cycle ESO; ^1^-^3^: Kruskal Wallis H value between first cycle ESO and baccalaureate; ^2^-^3^: Kruskal Wallis H value between second cycle ESO and baccalaureate.

**Table 3:** Bivariate correlations (Spearman) of the factors.

Variables	Intrinsic M.	Extrinsic M.	Perceived Quality	Satisfaction	Value of the Service
Intrinsic M.	1	0.596 **	0.099	0.057	0.133 *
Extrinsic M.	0.596 **	1	−0.057	−0.216 **	0.004
Perceived quality	0.099	−0.057	1	0.472 **	0.479 **
Satisfaction	0.057	−0.216 **	0.472 **	1	0.598 **
Value of the service	0.133 *	0.004	0.479 **	0.598 **	1

M.: motivation. Note: * (*p* < 0.05); ** (*p* < 0.01).

**Table 4:** Test coefficients for the AMPEF and EPOD2 questionnaire factors.

Dependent Variables	Independent Variables	β	T	*p*
Intrinsic M.	Extrinsic M.	0.620	13.801	<0.001
Extrinsic M.	Intrinsic M.	0.635	14.594	<0.001
	Satisfaction	−0.207	−4.743	<0.001
Perceived quality	Satisfaction	0.436	7.609	<0.001
	Value of the service	0.234	4.090	<0.001
Satisfaction	Value of the service	0.425	8.963	<0.001
	Perceived quality	0.361	7.613	<0.001
	Extrinsic M.	−0.0.143	−3.478	<0.001
Value of the service	Satisfaction	0.477	8.763	<0.001
	Perceived quality	0.223	4.090	<0.001

M.: motivation; β: Beta value; T: T-value; *p*: Statistical significance.

## Data Availability

Data available from the authors of this publication.
